# Plasmon induced modification of silicon nanocrystals photoluminescence in presence of gold nanostripes

**DOI:** 10.1038/s41598-018-22633-x

**Published:** 2018-03-20

**Authors:** S. A. Dyakov, D. M. Zhigunov, A. Marinins, O. A. Shalygina, P. P. Vabishchevich, M. R. Shcherbakov, D. E. Presnov, A. A. Fedyanin, P. K. Kashkarov, S. Popov, N. A. Gippius, S. G. Tikhodeev

**Affiliations:** 1 Skolkovo Institute of Science and Technology, Photonics, and Quantum Materials Center, Moscow, 143025 Russia; 2 Lomonosov Moscow State University, Faculty of Physics, Moscow, 119991 Russia; 3 KTH Royal Institute of Technology, School of Engineering, Stockholm, Sweden; 4 Skobeltsyn Institute of Nuclear Physics, Moscow, 119991 Russia; 5 National Research Centre Institute, Moscow, 123182 Russia; 6 A. M. Prokhorov General Physics Institute, Moscow, 119991 Russia

## Abstract

We report on the results of theoretical and experimental studies of photoluminescense of silicon nanocrystals in the proximity to plasmonic modes of different types. In the studied samples, the type of plasmonic mode is determined by the filling ratio of a one-dimensional array of gold stripes which covers the thin film with silicon nanocrystals on a quartz substrate. We analyze the extinction, photoluminesce spectra and decay kinetics of silicon nanocrystals and show that the incident and emitted light is coupled to the corresponding plasmonic mode. We demonstrate the modification of the extinction and photoluminesce spectra under the transition from wide to narrow gold stripes. The experimental extinction and photoluminescense spectra are in good agreement with theoretical calculations performed by the rigorous coupled wave analysis. We study the contribution of individual silicon nanocrystals to the overall photoluminescense intensity, depending on their spacial position inside the structure.

## Introduction

It is well known that a quantum dot placed in a strongly non-homogeneous dielectric environment can exhibit optical properties that are quite different from those of a quantum dot in free space^1–3^. From the optical point of view, the emission intensity depends on the excitation efficiency, photoluminescence out-coupling efficiency as well as on the probability of a quantum dot to radiate photons or to transfer its energy to the matrix nonradiatively^3^. The above parameters can be effectively tuned in the presence of metals. This fact is interesting from the viewpoint of potential optoelectronic applications of silicon nanocrystals given that silicon nanocrystals are CMOS-compatible and exhibit room-temperature photoluminescence^4–10^. Understanding of the mechanisms of interaction of silicon nanocrystals with metal is of great importance for design of optoelectronic devices based on Si quantum dots such as light emitting diodes where a homogeneous metal layer plays a role of an electrode^11–13^.

The effect of the influence of metallic nanostructures on the emission characteristics of nearby emitters have been observed in a variety of different molecules and quantum dots^3,14–28^. In particular, in ref.^17^, the authors studied the emission from silicon nanocrystals mediated by surface-plasmon polaritons (SPPs) in the homogeneous gold film. Photoluminescence of silicon nanocrystals in the presence of another type of plasmonic mode, namely, localized surface plasmons, was studied by Sugimoto *et al*. in ref.^19^. Authors demonstrated the triple enhancement of the quantum efficiency of the emission of silicon nanocrystals in colloidal nanocomposites containing gold nanorods. In refs^20,29,30^. Biteen *et al*. studied the emission of silicon quantum dots in the proximity of silver nanoparticles and demonstrated the photoluminescence intensity enhancement which was attributed to an increase in the radiative decay rate of the silicon quantum dots.

Despite the number of publications devoted to the optical properties of silicon nanocrystals in the proximity of metals are still of particular interest. In our recent paper^23^ we studied the photoluminescence of silicon nanocrystals near the localized surface plasmon modes supported by the periodic array of narrow gold nanostripes with fixed air slit between them, *w*. Depending on the air slit width, the gold nanostripes array can support surface plasmon-polaritons^31^ and (or) localized surface plasmons^32,33^. In this work, we vary the air slit width while keeping the array pitch size fixed and study how the parameter *w* affects the photoluminescence and extinction spectra of the samples. We describe the extinction and photoluminescence characteristics in terms of the optical resonances and calculate the electromagnetic near-field distribution at the resonance photon energies. Finally, we analyse the contribution of individual silicon nanocrystals to the overall PL intensity.

**Figure 1:**
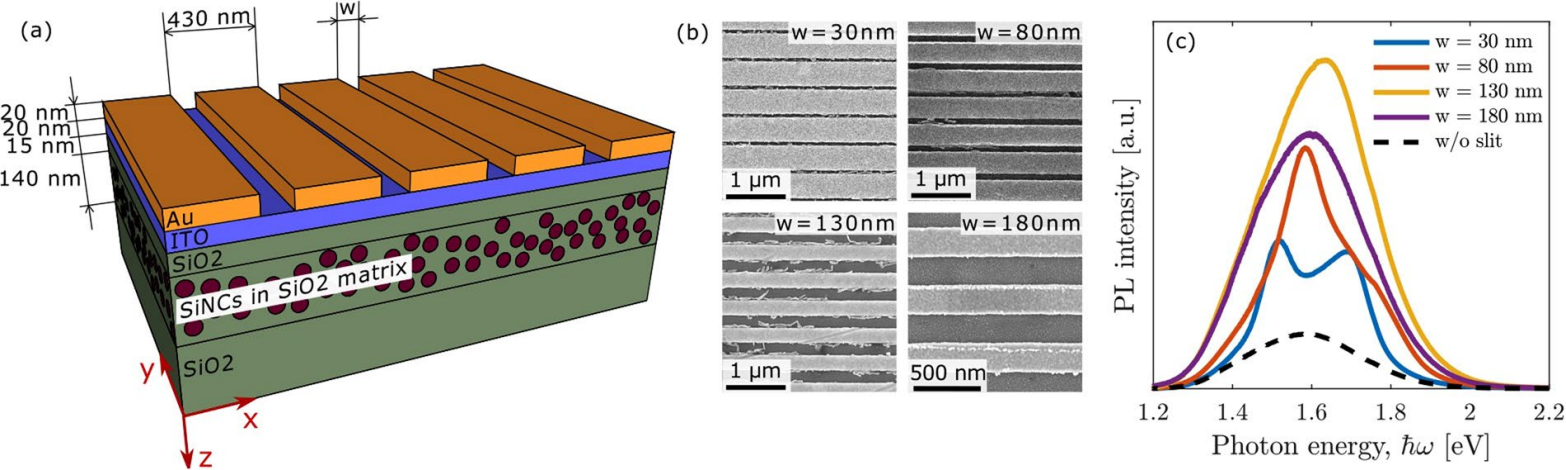
Sample and PL spectra. (**a**) Schematic view of the sample with silicon nanocrystals (shown by circles) and a gold grating. Silicon nanocrystals are shown by circles. (**b**) SEM images of structures under study. (**c**) Experimental PL spectra for different air slits widths. The black dashed line shows the spectrum for the uniform structure without slits.

The schematic of the investigated structure is shown in Fig. 1. The structure consists of an array of air slits in a 20 nm thick gold film deposited on a quartz substrate covered by thin SiO_2_ film with silicon nanocrystals. The air slits width was varied between *w* = 30 nm and 180 nm with a step of 50 nm. The pitch size was kept fixed at 430 nm. Silicon nanocrystals were evenly distributed in the SiO_2_ film on the depths from 35 nm to 175 nm. Scanning electron microscopy (SEM) images of the gratings with different air slit widths are shown in Fig. 1b.

The experimental PL spectra of the samples with different air slit widths are shown in Fig. 1c. One can see that for all *w* the PL intensity is higher than in the case of the sample covered by the gold film without air slits. It is remarkable that even narrow air slits (*w* = 30 nm which is less than 7% of the pitch size) give a raise of the PL intensity by a factor of 3 at some photon energies. The PL spectrum at *w* = 30 nm has two peaks. With the increase of the air slit width, the higher energy peak disappears. To understand the above behaviour of the PL spectra with the increase of the air slit width, in what follows we perform the detailed theoretical and experimental study of the optical resonances in our samples.

**Figure 2:**
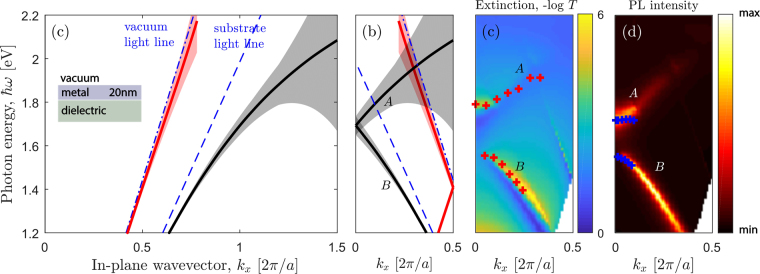
Dispersion relations of optical resonances. (**a**) Dispersion relation of SPPs (solid lines) of a uniform 20-nm thick gold plate on the dielectric substrate shown in the inset. Dashed lines correspond to vacuum and dielectric light lines. The width of shaded stripes denotes the imaginary part of eigenenergy at corresponding in-plane wavevectors. (**b**) The dispersions folded in the first Brillouin zone. (**c**) Extinction and (**d**) PL intensity as a two-dimensional function of photon energy and in-plane wavevector. Red and blue crosses represent the peaks in the experimental extinction and PL spectra measured for the 30-nm air slit sample respectively. The coloured scheme is explained on the color bar.

We start from the eigenmode analysis of our structure in the assumption of zero air slit width, *w* = 0. In this limit, the structure is in-plane uniform as shown in the inset of Fig. 2c. There are two surface plasmon polariton modes (see Fig. 2a) which correspond to metal-vacuum and metal-dielectric interfaces. Due to a small metal thickness, these plasmonic modes are coupled and this coupling changes their spectral position in comparison with the idealized case of semi-infinite metal. The width of the resonances is determined by the imaginary part of eigenenergy and is shown in Fig. 2a by shaded stripes. It can be seen that with the increase of the photon energy, the width of resonances grows due to the growing absorption of gold. In case of *w* > 0, the structure becomes periodic and the dispersion curves have to be folded into the first Brillouin zone (Fig. 2b). Each of surface plasmon polariton modes has now two branches which we denote as *A* and *B*. All the modes are shown in Fig. 2b are above the vacuum light line and hence are expected to be observed in the optical spectra of the sample with narrow air slits.

The calculated in-plane wavevector and photon energy dependencies of extinction (−logT) and out-coupling efficiency are shown in Fig. 2c,d for 30-nm air slit periodic structure. The experimental extinction and PL resonances are shown by red and blue crosses. By inspecting the Fig. 2c,d one can notice that the extinction and out-coupling efficiency have the features associated with the eigenmodes explained in Fig. 2b. Indeed, one can clearly see the resonances which are spectrally located close to the SPP modes. Besides of that, there are features associated with the vacuum and dielectric light lines folded into the first Brillouin zone. These are the Wood-Rayleigh anomalies which correspond to the opening of new diffraction channels. In our experiment, the Wood-Rayleigh anomalies are not observed because their spectral positions are outside of the detection range of the experimental setup.

## Results and Discussion

**Figure 3:**
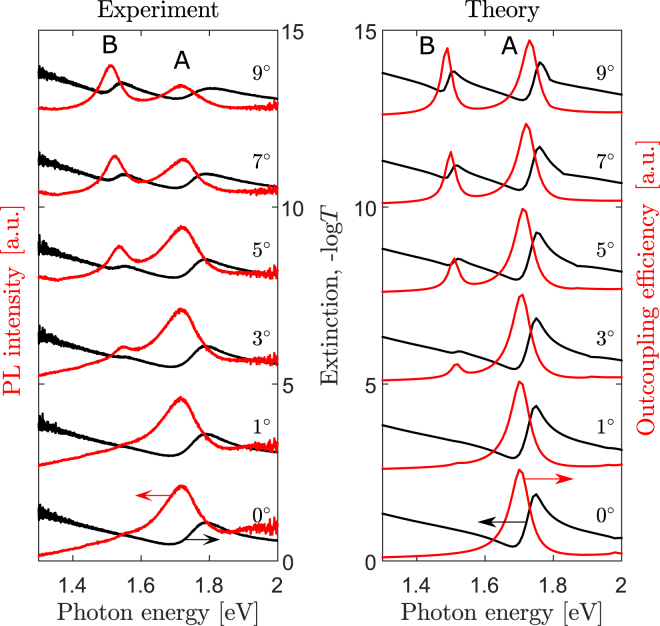
Extinction and PL spectra. (**a**) Experimental extinction (black lines) and PL spectra (red lines). The displayed angles denote the angle of light incidence. (**b**) Theoretical extinction (black lines) and out-coupling efficiency spectra (red lines). The displayed angles denote the angle of PL collection. Curves in panels (**a**) and (**b**) are shown for the sample with 30-nm-wide air slits.

For more details, let us consider the angle-resolved TM-polarized experimental extinction and unpolarized PL spectra, as well as their theoretical counterparts (Fig. 3a). The experimental PL spectra are normalized to the reference spectrum of the sample without the gold layer. Several important features in the peaks behaviour can be seen in Fig. 3. First, both extinction and PL spectra have two series of peaks *A* and *B*. With the increase of the observation angle, the spectral distance between the peaks increases. This is well correlated with calculated surface plasmon polariton mode dispersions. As can be seen from Figs 2 and 3, at small in-plane wavevectors, the upper and lower surface plasmon polariton branches in the optical spectra are coupled and their spectral positions deviate from the dispersion curves calculated for *w* = 0. At larger observation angles (*θ* ≳ 20°) the upper surface plasmon polariton branch is not so pronounced in the spectra due to the large width of this resonance as predicted by the imaginary part of eigenmode energy.

**Figure 4:**
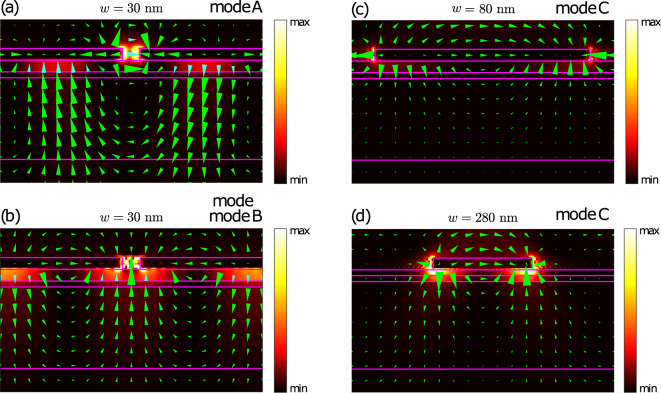
Electric field distributions. Calculated spatial distributions of the electric field in the structure with different air slit widths *w* for normal incidence of TM polarized light. The fields are shown for the photon energy of (**a**) *ћω* = 1.72 eV, (**b**) *ћω* = 1.53 eV, (**c**) *ћω* = 1.24 eV, (**d**) *ћω* = 1.57 eV. The size of triangles is proportional to the field at the central point of each triangle. Triangles specify the corresponding electric field direction by their orientation. The electric field represented by cyan triangles, it is reduced by a factor of three compared to blue triangles, to prevent the triangles overlap. Background color shows the electric field intensity as explained in the color bars.

The second important feature of the extinction and PL spectra is that at zero observation angle, the spectra have only one peak which corresponds to the upper SPP branch. The lower SPP peak appears at inclined observation angle *θ* > 0. Such behaviour is explained by the modes symmetry which can be understood by inspecting the electric near-field distributions of the corresponding eigenmodes at *k*_*x*_ = 0 (see Fig. 4). Figure 4 shows the electric field distributions of the eigenmodes at the photon energies *ћω* = 1.72 eV and 1.53 eV which correspond to the modes *A* and *B*. At both photon energies, the displayed field decays into the superstrate and the substrate. The electric field at *ћω* = 1.53 eV represents the symmetric lower branch surface plasmon-polariton mode as shown in Fig. 4a. Due to its symmetry, this mode is optically inactive at *k*_*x*_ = 0 and hence can only be observed in extinction and photoluminescence spectra under an inclined incidence. In contrast, the mode at *ћω* = 1.72 eV takes the shape of vortices and is antisymmetric (Fig. 4b) which makes it visible under any angle of incidence.

The third feature of the spectra displayed in Fig. 3 is that the extinction and PL resonances have different shapes. The extinction spectra have a characteristic asymmetric Fano line shape since they are originated from discrete SPP modes on the background of the photon continua in the air and the substrate^34–36^. In the PL spectra, the resonances do not interfere with the photon continua and hence are described by a simple Lorentz profile.

It is worth mentioning that our theoretical extinction and out-coupling efficiency spectra (Fig. 3b) agree with the experimental results.

### Plasmonic resonances in structures with silicon nanocrystals

**Figure 5:**
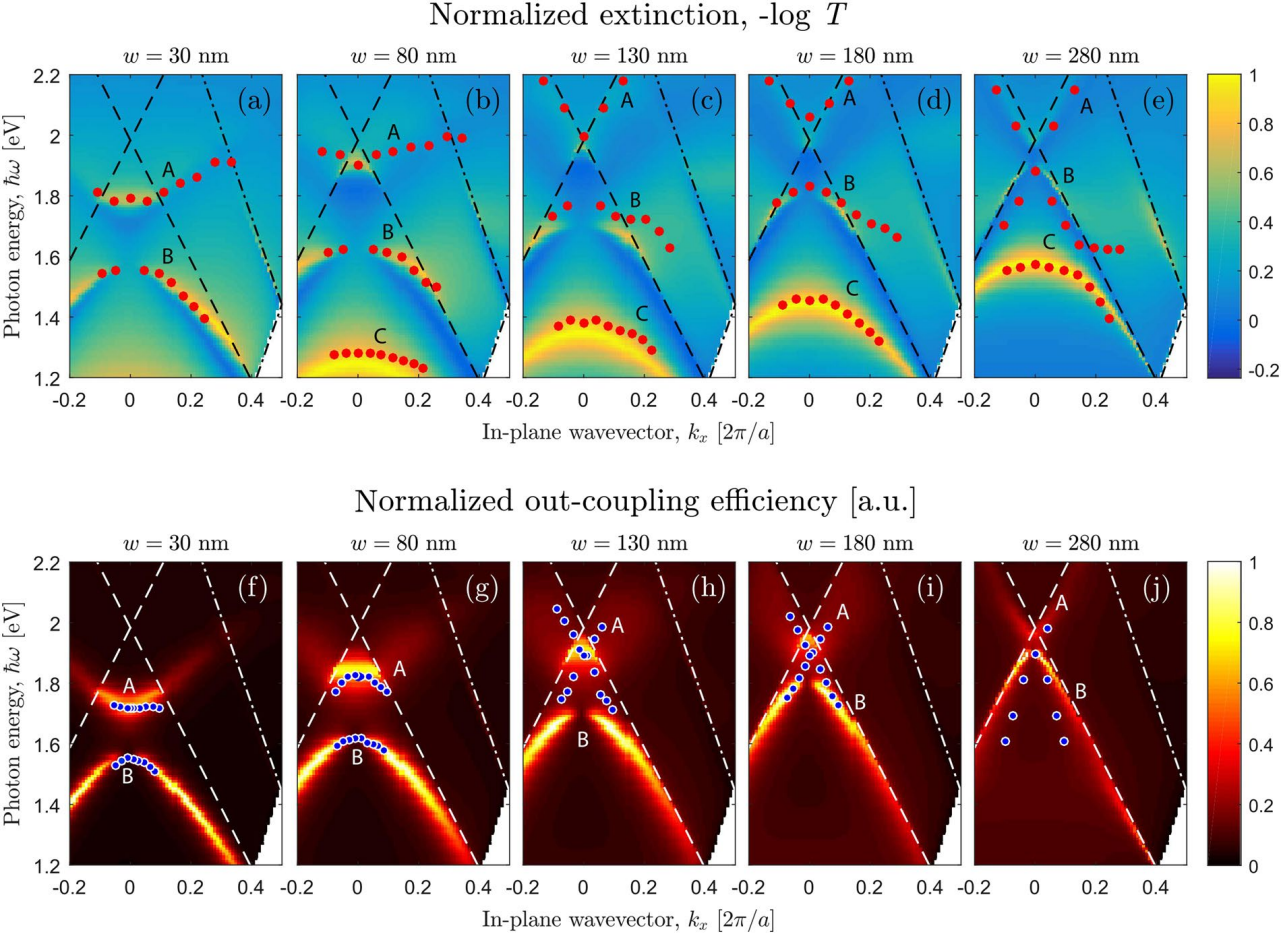
Extinction coefficient and out-coupling efficiency as functions of in-plane wavevector and photon energy. (**a**) Calculated *k*_*x*_ and energy dependencies of the extinction (panels (a)–(e)) and emissivity (panels (f)–(j)) spectra of the TM-polarized light for the sample with different air slit width. The magnitude of the extinction coefficient and emissivity is shown by different colors and the color scale is explained in color bars on the right. The red dots in panels (a)–(e) represent the experimentally observed extinction peaks, while the blue dots in panels (f)–(j) show the experimental PL peaks. The peak positions for the sample with 280 nm air slit are taken from ref.^23^.

Let us now study the behaviour of the extinction and photoluminescence spectra under the transition from narrow to wide air slits. For this purpose, we calculate the in-plane wavevector and energy dependence of the extinction and out-coupling efficiency for *w* changing from 30 nm to 280 nm (Fig. 5a–j). With the increase of the air slit width, the upper and lower branches of SPP mode, *A* and *B*, are transformed into the quasiguided modes. As was shown in ref.^23^, the quasiguided modes are localized in the layer with silicon nanocrystals which plays the role of a waveguide. Such modes are seen in the extinction spectra due to the structure periodicity^37^. Besides of the modes *A* and *B*, there is the mode *C* which falls within the measurement range at *w* > 80 nm (see Fig. 5b). The electric field of this mode (see Fig. 4c,d) is localized near the gold stripe edges and has the characteristic shape of a dipolar resonance located on the gold stripes. Thus, the mode *C* represents the localized surface plasmon (LSP) resonance. With increase of air slit width *w*, the mode *C* blueshifts and interacts with the lower quasiguided mode *B*. Owing to the strong coupling between the LSP mode *C* and the quisiguided mode *B*, a hybrid mode of waveguide plasmon polariton appears^32,33^ yielding in enhancement of PL intensity at corresponding wavelengths. This hybrid mode has two branches *B* and *C* and the Rabi splitting between them is about 100 meV.

In contrast to the extinction spectra, the out-coupling efficiency shows only two sets of modes, *A* and *B*. It should be noted that the LSP mode *C* is not seen in the photoluminesce spectra because it is localized on the metal edges and hence is highly absorptive. The experimental extinction and PL peaks positions (shown by circles in Fig. 5) are well described by our theoretical model.

As a result, the above analysis of optical resonances in the sample in study explains the features of the experimental PL spectra shown in Fig. 1c. Indeed, as can be seen in Fig. 5, the structure with 30-nm-wide gold grating is characterized by almost dispersionless PL peak behaviour that explains the visibility of two separate peaks in the angle-integrated PL spectra. For large air slit width, the dispersion of the optical resonances becomes stronger which results in single wide peaks for *w* > 80 nm in the angle-integrated PL spectra.

**Figure 6:**
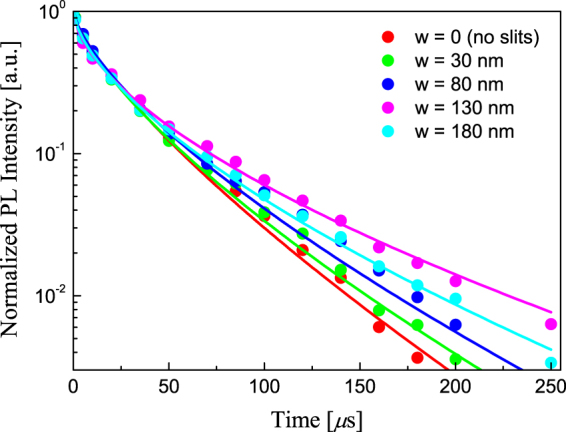
PL decays. PL decay curves with variable air slit width *w* for *ћω* = 1.6 eV. Dots are experimentally detected PL intensities, lines are stretched exponentials functions fitted to experimental data.

We have also studied the effect of the air slits on the PL lifetime by measuring time-resolved PL spectra. The obtained PL decays are subjected to a standard stretched exponential fitting procedure: *I*(*t*) = *I*_0_ exp{−(*t*/*τ*)^*β*^}, where *τ* is a PL lifetime and *β* is a nonmonoexponentiality parameter. The experimentally obtained PL decays and the corresponding fitted curves for the photon energy of 1.6 eV are shown in Fig. 6 for different air slit widths *w*. It can be seen that the decay profile depends on the parameter *w* nonmonotonically. The comparison between the PL lifetimes of the samples with and without gold reveals that neither a gold grating nor a slit-free homogeneous gold layer (*w* = 0) changes the PL relaxation dramatically. Indeed, PL lifetime in Fig. 6 varies in the range between 17.5 and 19.0 *μ*s. This is in agreement with the fact that in our sample the closest distance between nanocrystals and metal is 25 nm, which excludes a strong photoluminescence quenching on lossy metallic modes. Nevertheless, with increase of the air slit width, the plasmonic and waveguided modes are transformed and thereby affect the PL lifetime that explains the small variations of the PL lifetime with air slit width.

### Contribution of individual dipoles to overall PL intensity

In the previous section, the out-coupling efficiency of the samples in study was calculated as an integral over all emitter positions within the layer with silicon nanocrystals. At the same time, it is obvious that silicon nanocrystals in different positions inside the structure are in different optical conditions. From the viewpoint of expression for the overall PL intensity (2), it means that the excitation efficiency ${|{\bf{E}}(\hslash {\omega }_{exc},{{\bf{k}}}_{||exc},{{\bf{r}}}_{i})|}^{2}$ as well as the out-coupling efficiency ${|{\bf{E}}(\hslash {\omega }_{pl},{{\bf{k}}}_{||pl},{{\bf{r}}}_{i})|}^{2}$ both depend on the emitter position **r**_*i*_. The spatial non-uniformity of the excitation efficiency indicates that the concentration of excited silicon nanocrystals in one part of the sample is higher than in the other. The spatial non-uniformity of PL is additionally modified by that of the out-coupling efficiency. As a result, silicon nanocrystals that are located in different positions within the active layer, give a different contribution to the overall PL intensity.

**Figure 7:**
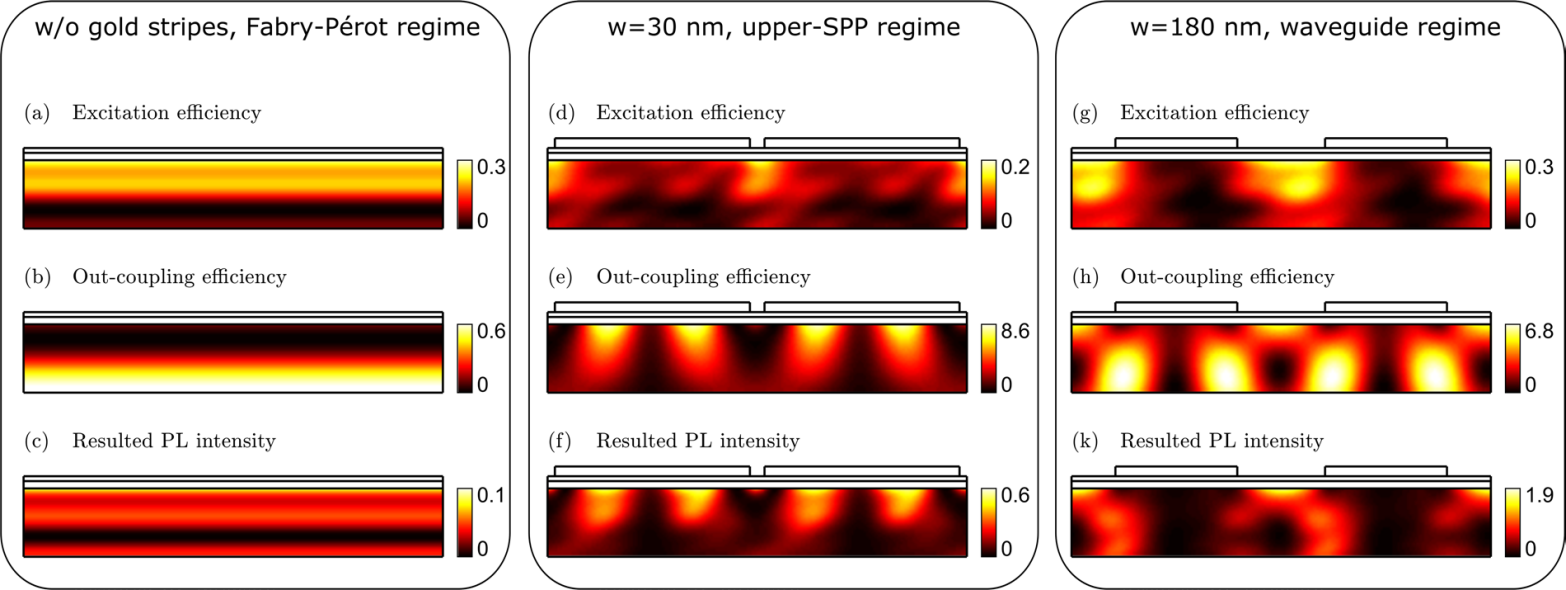
Spatial distributions of excitation efficiency, out-coupling efficiency and resulted PL intensity. Calculated excitation efficiency (**a**),(**d**),(**g**), outcoupling efficiency (**b**),(**e**),(**h**), and resulted PL intensity as a function of dipole position within the layer with silicon nanocrystals (**c**),(**f**),(**k**). The color scales of the corresponding quantities are shown in the color bars on the right. For comparison, the above quantities are shown for *w* = 0, 30, and 180 nm. Quartz substrate is not shown in panels (**a**)–(**k**).

The calculated excitation efficiency and the outcoupling efficiency as functions of the emitter position are shown in Fig. 7 for three different regimes. In the discussion below, all the structures are exposed by 325 nm laser at *θ*_*exc*_ = 45° angle of incidence, a typical excitation scheme in our experimental setup. We consider first the reference structure without gold layer. The photoluminescence is detected at 1.6 eV (the PL peak energy of silicon nanocrystals) at the normal collection angle. It can be seen from Fig. 7a that the excitation field is mainly localized in the sub-surface region causing the inhomogeneous profile of the excited silicon nanocrystals concentration. The spatial dependence of the outcoupling efficiency in the reference structure is shown in Fig. 7b. Notably, for this particular structure and experimental conditions, the highest probability for the emitted photons to escape the structure is localized deep inside the emitting layer. The distributions of excitation and outcoupling efficiencies are determined by the Fabry-Pérot resonances. The resulted PL intensity is found as a product of excitation efficiency and outcoupling efficiency and is displayed in Fig. 7c. It can be seen that in the reference structure, the excitation and outcoupling efficiency maps have a little overlap which leads to moderately low overall PL intensity. By changing the thicknesses of layers one can design the structure in such a way that excitation and out-coupling profiles match each other yielding in effectively enhanced PL signal^38^.

Let us consider the 30-nm-width air slit structure. The photoluminescence is detected at a photon energy of 1.72 eV at the normal collection angle, which corresponds to the upper SPP branch (see Fig. 5a). It can be seen from Fig. 7d that the excitation field is mainly located close to the air slits. The outcoupling efficiency is determined by the electric field distribution of the upper SPP branch and is shown in Fig. 7e. Since this mode has a standing-wave character, the outcoupling modulation coefficient, i.e., the ratio of the minimal and the maximal outcoupling efficiency within the emitting layer, is rather high. The resulting PL efficiency for the 30-nm-width air slit structure is shown in Fig. 7f. The displayed profile of the PL efficiency suggests that the major contribution to the overall PL intensity is brought by the nanocrystals located under the gold stripes in accordance with field distribution of the upper SPP branch.

Finally, we turn to the 180-nm-width air slit structure. The excitation field is maximal in the regions underneath the slits (Fig. 7g). In the calculation, we choose the photoluminesce photon energy to match the upper quasiguided branch at the normal collection angle (*ћω* = 1.96 eV). Figure 7h reveals that the outcoupling efficiency takes the shape of loops which is dictated by the field distribution of quisiguided modes^23^. Likewise in ref.^23^, due to the quasiguided modes, the photoluminesce of such structure is enhanced and the outcoupling modulation coefficient for this structure is very high (≈550). This indicates the strongly non-uniform distribution of silicon nanocrystals contribution to the resulted PL signal Fig. 7k).

The above excitation and outcoupling efficiency distributions over the ensemble of silicon nanocrystals represent purely optical effects and may be smoothed in reality due to the exciton migration process. Nevertheless, the high amplitude of the outcoupling modulation coefficients allows us to to separate the possible nanocrystal locations into optically bright and optically dark depending on the magnitude of their contribution to the overall PL efficiency. It should be noted that such separation depends not only on the sample geometry but also on the experimental conditions, i.e., on the excitation and PL wavelengths, polarizations, excitation angle, and collection angle. The regions of optically dark nanocrystals locations can be seen in Fig. 7c,f,k as black spots, while optically bright locations are in red and yellow areas. Since, generally speaking, the silicon nanocrystals PL lifetime depends on the pump power, the only fact of the high modulation in the excitation efficiency profile (Fig. 7a,d,g) might lead to different PL lifetimes of optically bright and optically dark nanocrystals. Besides, the non-homogeneous distribution of the Purcell factor within the layer with silicon nanocrystals can additionally influence the PL lifetimes of the dark and bright subensembles. (Due to the risk of sample damage by a high intensity of the laser beam, we performed the PL decay measurements at very low excitation power. Hence, we do not expect to observe the phenomenon of bright and dark nanocrystals in our experiment).

## Conclusion

In conclusion, we have theoretically and experimentally studied the optical properties of silicon nanocrystals covered by periodic arrays of gold stripes. We have shown that the extinction and photoluminescence spectra have several sets of peaks, which are attributed to surface plasmon-polaritons, localized surface plasmons or quasiguided modes depending on the air slit width. We have also shown the transition between these modes and their impact on the PL lifetime with the increase of the air slit width. Finally, we have analysed how the position of a silicon nanocrystal within the structure affects its contribution to the overall PL intensity. We found that in the surface plasmon-polariton regime, the major contribution to the PL intensity comes from the sub-surface silicon nanocrystals. In the waveguide regime, when air slit width is large, the PL is contributed by silicon nanocrystals in depth of the emitting layer. In both cases, the distribution of silicon nanocrystals contribution to the overall PL intensity is highly non-uniform.

## Methods

### Sample and experimental details

Reactive evaporation of SiO powder in an oxygen atmosphere was used to deposit SiO_*x*_ (*x* ≈ 1.7) films on quartz substrates. Films thickness was equal to about 140 nm, while a capping 15 nm thick SiO_2_ layer was also deposited on a top of the structure under study by increasing an oxygen pressure during evaporation. After the deposition the conventional tube furnace annealing at 1100 °C for 1 hour in N_2_ atmosphere was used in order to fabricate Si nanocrystals in SiO_2_ matrix (see for details ref.^39^).

For the gold nanostripe fabrication, the sample was additionally covered with 10-nm thick indium tin oxide (ITO) layer as a transparent adhesion promoter between gold and silica. Then, the sample was cleaned, CSAR 62 resist spin coated, and baked forming a 140 nm thick uniform layer. Next, 500 × 500 *μ*m gratings were patterned with electron beam lithography system (Raith 150, 25 kV acceleration voltage) using fixed beam moving stage (FBMS) mode. This allowed uniform exposure. After development, the 20 nm of gold film was deposited in the high vacuum e-gun evaporation system (Eurovac). Then, the lift-off process was performed by immersing the sample in acetone. This removed the photoresist with the excess Au leaving only Au grating lines deposited on ITO.

The SEM images were captured by a high-resolution Field Emission Scanning Electron Microscope Supra 40 (Carl Zeiss).

Transmittance spectra were measured as a function of the angle of light incidence. In the setup, light from a broadband source (50 W halogen lamp) is collimated and slightly focused to a spot of about 500 *μ*m in diameter. The polarization state is controlled by a Glan-Taylor polarizer. The transmitted beam is collected and sent to a compact CCD-based visible spectrometer. The sample is held by a 3-axis holder that allows for the control of the incidence angle in XZ plane with a step of 1°. The spectra were measured consecutively for the sample area and the substrate without gold grating; then, the sample spectra are normalized over the substrate spectra.

Photoluminescence (PL) spectra were registered under the 325 nm HeCd laser line excitation using 500 mm single-grating spectrometer equipped with an air-cooled CCD camera. The spectra were taken at room temperature and were corrected for the system response.

In order to obtain silicon nanocrystals PL relaxation characteristics, time-resolved PL spectra were first measured using a pulsed Nd:YAG laser excitation (wavelength 532 nm, 10 Hz repetition rate, 34 ps pulse duration, laser pulse fluence ~3 mJ/cm^2^). The PL signal was collected by means of intensified CCD (PI-MAX Gen III, Princeton Instruments) coupled to a 500 mm focal length imaging spectrograph (SpectraPro 2500i, Princeton Instruments). All PL spectra were detected within 1 *μ*s gate width taking various delays after excitation pulse onset in the range from 0 to 250 *μ*s. The PL decays for different air slit widths were plotted using the PL intensity at a chosen emission wavelength as a function of delay time.

### Theoretical methods

The photoluminescence intensity was calculated as a power emitted by the oscillating electric dipoles uniformly and randomly distributed over the layer with silicon nanocrystals. From the population dynamics equations for silicon nanocrystals (see, for example, ref.^30^) it follows, that in the approximation of low excitation power, the emission intensity of single dipole is proportional to the product of excitation efficiency *C*_*exc*_ and the out-coupling efficiency *C*_*out*_:

1 $${I}_{i}\propto {C}_{exc}{C}_{out}.$$

The parameters *C*_*exc*_ and *C*_*out*_ are large in the spectral vicinity of plasmonic modes due to the resonant field enhancement. The overall PL intensity accounts for the contribution from uniformly distributed dipole sources:

2 $$I=\sum _{i}{I}_{i},$$

where emission intensity of *i*-th SiNCs in the ensemble is given by the formula (1) for the general case. The contribution of each dipole source, *I*_*i*_, can be calculated as

3 $${I}_{i}\sim {|{\bf{E}}(\hslash {\omega }_{exc},{{\bf{k}}}_{||exc},{{\bf{r}}}_{i})|}^{2}\times {|{\bf{E}}(\hslash {\omega }_{PL},{{\bf{k}}}_{||PL},{{\bf{r}}}_{i})|}^{2},$$

where **E** is the electric vector of incidence plane electromagnetic wave calculated at the photon energy *ћω*_*α*_, the in-plane projection of the photon quasimomentum vector ${{\bf{k}}}_{||\alpha }\equiv ({k}_{x\alpha },{k}_{y\alpha })$, and the coordinate of oscillating dipole **r**_*i*_ ≡ (*x*_*i*_, *z*_*i*_). The symbol *α* = “*exc*” or “*PL*” relates to the excitation or photoluminescence. The first factor in Eq. (3) is the excitation efficiency *C*_*exc*_; it is proportional to the volume density of excited nanocrystals at the position **r**_*i*_. The second factor in Eq. (3), in accordance with the electrodynamic reciprocity principle has the meaning of an out-coupling efficiency *C*_*out*_ which is proportional to the probability for the emitted photon to come out from the sample and couple to the far field. Summation over PL intensities from different nanocrystals in Eq. 2 means that we consider all dipoles to emit incoherently which excludes interference effects between different dipoles.

Calculations of the electric field *E* are performed using the rigorous coupled wave analysis (RCWA) in the scattering matrix form^37,40,41^. The general idea of this method is the Fourier decomposition of the electromagnetic field into planar waves with different projections of the momentum vector onto the direction of periodicity. In order to achieve a better convergence with respect to the number of plane waves, we employ the Li’s factorization rules^42^.

From the scattering matrix we can calculate the optical reflection and transmission coefficients. We can also determine the structure eigenmodes by solving the eigenvalue problem for the inverse scattering matrix ${\mathbb{S}}$:

4 $${{\mathbb{S}}}^{-1}(\omega ,{{\bf{k}}}_{||})|O\rangle =\mathrm{0,}$$

where the |*O*〉 is output amplitudes vector which describes the eigenmode (see ref.^37^ for details).
